# Ungrateful Patients

**Published:** 1892-02-20

**Authors:** 


### THE DOCTOR'S ARMCHAIR.

### UNGRATEFUL PATIENTS.

Is there any reason why, if a patient pays a doctor his bill, he should give him gratitude as well? Why should one be grateful to one's doctor any more than to one's butcher? But the present writer has nothing whatever to say against being grateful to his butcher. On the contrary, when his butcher regularly supplies him with good meat at a reasonable price, and throws in a proper measure of civility with it, the butcher has a right to hope, not only that his bill will be punctually paid, but that it will be paid pleasantly, and even gratefully. The butcher has done all that he was required to do, and he has done it well; but he has also done more; he has made the buying of meat a pleasure as well as an advantage. Therefore, a well-conditioned man will say, "Thank you, Mr. Sirloin," and he will say it heartily.

On this line of reasoning it is easy and just to claim gratitude for the doctor. Given a doctor who does his work thoroughly well, and with a pleasant manner, and who charges for it a just and reasonable fee, there are many excellent reasons why a patient who is the recipient of such a doctor's trained skill should not only pay his account promptly, but should also feel and express towards the doctor deep and heartfelt gratitude. This, in fact, is quite universally recognised. The wholesome-hearted doctor, particularly the family physician, is looked upon as a kind of hero. The present writer knows one old gentleman, nearly seventy years of age, who devotes himself to his patients with all the affection of a father, and all the trained skill of an accomplished medical practitioner. To his patients, old and young alike, that old gentleman is the embodiment of every excellence that is to be found in the law and in the Gospel. So far from any of his patients finding it difficult to be grateful to Mr. —— —for he is not an M.D.—they would find it not only difficult but impossible to feel anything for him but gratitude and love. Bless the old gentleman! He sweetens and brightens every house he enters by the wholesomeness of his character, and the skill and success of his work.

But a doctor, that is, of course, a worthy doctor, has really much more claim to gratitude than the honest tradesman, or than, indeed, almost any other person whose life is spent in the service of the public. It is true he charges his fee, and sends in his "little bill." But then it is a very "little" bill, indeed. Moreover, when he is doing his work he never thinks of his bill; he gives himself up heart and soul to the helping, comforting, and saving of struggling men and women in the hour of their most desperate need. Many and many a time he makes his visit twice, or even thrice, its ordinary length, without so much as thinking of doubling or trebling his fee, or even of adding so much as a single sixpence to it. Such services as the really good doctor renders are never paid for, and cannot be paid for in money. A certain cynical person divided human beings into the three sexes of men, women, and the "clergy," thinking thereby to cast a little slur upon the clergy. As a matter of fact, the best of the clergy are better than either men or women separately, for thay combine in themselves the worthiest character elements of both the sexes. The same is emphatically true of the best doctors. They may not be keen financiers, or pushing commercial men, or ambitious parliamentarians, or brilliant writers. But they may be and often are, in the worthiest analysis, a thousand times better and greater than any of those energetic people. They are saints without theology, and angels with solid bodies living in an earthly sphere.

What is the matter with the Doctor's Armchair this week? some curious reader may ask. Nothing is the matter with the Doctor or his Armchair; but an instance of base ingratitude on the part of a patient towards a competent doctor is reported in the public newspapers: and it is so gross a case in every way that no honest medical man can help feeling a good deal of indignation. Besides, in these columns we are so often exhorting the members of the medical profession to aspire after a high standard of duty towards their patients, that it is but fair we should sometimes take a look at the other side of the shield and encourage patients a little to aim at a high standard of duty towards their doctors. The case indicated was "one of a claim on the part of Dr. Batchelor, the plaintiff, for a fee for medical attendance; and a counter-claim on the part of his patient, who was the defendant, for damages for alleged unskilful treatment. Mr. Gore, a contractor, aged 55, after having suffered during four years from pulpy disease of the right knee, for which he was first treated by Dr. Batchelor, and subsequently on and off by other practitioners, returned to the plaintiff in September, 1888, deteriorated in health and with the diseased joint much disorganised. The plaintiff, convinced that it would have been useless to persevere with expectant treatment, in which opinion he was supported by other experienced surgeons, excised the diseased joint. The age and unhealthy condition of the defendant being considered, it seems to have been a somewhat bold step on the part of Dr. Batchelor to undertake excision in this case. Nothwithstanding the unfavourable conditions, however, the correctness of his view was justified by the results, as it was shown by strong medical testimony, and also by the admissions of Mr. Gore himself, that the operation had been followed by complete success. The defendant in cross-examination, acknowledged that he was able with his saved limb to descend steep embankments, to attend racecourses, and to visit mines. He is to be seen daily, it is stated, walking about the streets with a light stick, apparently quite restored to health, and able to follow his usual avocations. After a brief consultation a verdict of three-fourths of the jury was returned for the plaintiff, awarding the full amount claimed."

Now, if this defendant, Mr. Gore, is to be tried by the old standard of "doing as one would be done by," then undoubtedly he served his skilful doctor a mean and disgraceful trick. The facts, briefly recapitulated, are that Dr. Batchelor was Mr. Gore's medical attendant at the outset; that Mr. Gore changed his doctor several times, but grew rather worse than better in consequence; and that, finally, he returned to Dr. Batchelor, who made a bold and skilful cure of a very unpromising case. For all this his reward was to be refused his fees, and to be charged with unskilful practice. The only possible ground for such a charge was, that some other surgeon might have selected a different operation from that actually performed by Dr. Batchelor. But if every surgeon who selects one of three or four possible operations is to be charged with want of skill, because some other surgeon would not select that particular operation but another, the result will be that no surgeon will select any operation at all. It will not then be the surgeon who will suffer, but the patient. If surgeons are to be hampered in the choice of their methods by the possibility that one patient in every ten or twelve will be down upon them for damages, whether they succeed or whether they fail, thousands of lives will be sacrificed every year for want of surgical operations. Mr. Gore failed in his charge against Dr. Batchelor, and the jury awarded the doctor the full amount of his fees. But if the jury had been stupid, or prejudiced against doctors, as many juries are, Mr. Gore would have received all Dr. Batchelor's skilful treatment for nothing, and would have had the gratification of ruining the doctor into the bargain. A medical man holds a position of great delicacy, and of painful responsibility. It is the duty of his patients to stand by him loyally, and not only their duty, but their interest. Trust him; give him a free hand: sympathise with his difficulties; be grateful for his skill and devotion. These are the rules which draw from the doctor all that is worthiest in his character, and all that is most skilful and efficient in his professional work.

